# Investigating the effects of increasing O‐GlcNacylation in Alzheimer’s disease in the TgF344‐AD rat model

**DOI:** 10.1002/alz.093507

**Published:** 2025-01-03

**Authors:** Melissa L Garcia, Lori L McMahon, Nateka L Jackson

**Affiliations:** ^1^ The University of Alabama at Birmingham, Birmingham, AL USA; ^2^ The Medical University of South Carolina, Charleston, SC USA

## Abstract

**Background:**

Alzheimer’s disease (AD) pathology can start accumulating 20‐30 years before cognitive symptoms occur, with increases in inflammation, amyloid‐β (Aβ), and hyperphosphorylated Tau during this time. Previous studies have shown that the post‐translational modification of a single N‐acetylglucosamine moiety to serine or threonine residues to cytosolic or nuclear proteins, known as O‐GlcNAcylation, can modify a plethora of cellular processes, including the processing of the amyloid precursor protein, competing with phosphorylation on tau, as well as having anti‐inflammatory effects. This study is designed to evaluate how increasing O‐GlcNAcylation is impacting AD pathology in the most comprehensive AD rat model to date, the TgF344‐AD rat model.

**Method:**

For these experiments, we used 6‐month‐old TgF344‐AD (**Tg**) rats and non‐transgenic (**nTg**) littermates injected with 10mg/kg of thiamet‐G (**TMG**), an inhibitor of O‐GlcNAcase, (**nTg‐TMG** or **Tg‐TMG)** or saline (**nTg‐S** or **Tg‐S**) as a control, 3 times a week from 6‐9 months. We then sacrificed the animals via cardiac perfusion with oxygenated artificial cerebrospinal fluid before splitting the brain in half; one hemisphere to be drop fixed in PFA for immunohistochemistry and half to be sub dissected and flash frozen for protein analysis.

**Result:**

We have preliminary results that confirm significant increases in GFAP, Iba1 and Amyloid‐β in Tg‐S rats compared to nTg‐S rats, however GFAP and Iba1 protein levels in Tg‐TMG rats are no longer significantly different from nTg‐S rats after 3 months of TMG injections to increase O‐GlcNAcylation. We have also evaluated noradrenergic innervation into the dentate gyrus using anti‐tyrosine hydroxylase (**TH**) immunohistochemistry and confocal imaging to evaluate if increasing O‐GlcNAc protects against loss of TH innervation of the dentate gyrus, and have so far confirmed our previous results at this time point; TMG treated animals are not significantly different from their saline controls.

**Conclusion:**

We report that increasing O‐GlcNAcylation from 6‐9 months in the TgF344‐AD rats is reducing reactive astrocytes. While this could be beneficial to preserving their function and neuronal health, this work is still ongoing. We are working to increase our sample size, and to examine the locus coeruleus for changes in hyperphosphorylated tau and other biomarkers.

